# Spiclomazine displays a preferential anti-tumor activity in mutant *KRas*-driven pancreatic cancer

**DOI:** 10.18632/oncotarget.24025

**Published:** 2018-01-08

**Authors:** Xiaoyu Guo, Wenjing Zhao, Zuojia Liu, Jin Wang

**Affiliations:** ^1^ State Key Laboratory of Electroanalytical Chemistry, Changchun Institute of Applied Chemistry, Chinese Academy of Sciences, Changchun, Jilin, China; ^2^ Department of Chemistry and Physics, State University of New York, Stony Brook, New York, USA

**Keywords:** pancreatic cancer, Ras, spiclomazine, targeted therapy, xenograft model

## Abstract

Ras-targeted therapy represents a ‘holy grail’ in oncology. Based on our model prediction, Spiclomazine freezing the intermediate conformation of activated Ras is central to cancer therapeutics. We show here that Spiclomazine leads to an effective suppression in Ras-mediated signaling through abrogating the KRas-GTP level in the *KRas*-driven pancreatic cancer. The Ras-mediated signaling inhibition leads to dramatically reduced survivals of five *KRas*-driven pancreatic cancer cell lines with IC_50_ ranging 19.7~74.2 μM after 48 hours of treatment. However, no significant changes have been observed for normal cell lines. It is worth mentioning that the mutant *KRas*-driven cancer cells are more sensitive towards Spiclomazine than the wild-type *KRas* cancer cells. Subsequent cellular thermal shift and RNA interference assays show that Spiclomazine efficiently binds with and stabilizes KRas to a certain extent within the cells. This validates the effect of target engagement on drug efficacy. Furthermore, Spiclomazine arrests cell cycle at G2 phase in the cancer cells, without obvious cell-cycle arrest in the normal cells. This further demonstrates its selectively biological response to cancer cells involved in Ras-GTP-mediated target engagement. Spiclomazine completely inhibits the growth of MIA PaCa-2 tumors on renal capsule xenograft models in BALB/c mice administered 68 mg kg^−1^ for 2 weeks via intra-peritoneal route. Immunohistochemical analyses reveal the reduced c-Raf and p-ERK and the increase in TUNEL staining. These observations further confirm the *in vitro* findings. Taken together, Spiclomazine is a selective inhibitor for mutant *KRas*-driven pancreatic cancer.

## INTRODUCTION

Pancreatic cancer, accounting for about 3% of all cancers, is becoming the 3rd leading cause of cancer-related death in the US [1]. Although current therapeutic strategies for the treatment of pancreatic cancer have made some progresses, the average 5-year survival rate is now 9%, moving up from the previous 8%, which has reached a plateau [2]. Of all the potential theranostic biomarkers of pancreatic cancer, mutations in the *KRas* genes are characterized by their particular frequency, approximately nine of tenth. Mutations in the other *Ras* isoforms (*NRas* or *HRas*) have been described in pancreatic cancer, though these are unusual ones (<2%) [3]. The definite relations between mutations in the *KRas* proto-oncogenes and the tumorigenesis of pancreatic cancer make KRas one of the more attractive drug targets.

Although Ras is considered to be “undruggable”, several research groups have developed direct Ras inhibitors. The common mechanism of direct binding to and inhibiting mutant KRas involves blocking GEF-catalysed nucleotide exchange [4]. Walensky and colleagues designed a molecule which blocked nucleotide association with KRas *in vitro* and decreased the viability of the mutant *KRas*^G12D^ pancreatic cancer cells with an IC_50_ of ~10 μM [5]. Palmioli A et al reported a bicyclic Ras inhibitor which decreased the viability of the mutant *KRas*^G13D^ colorectal carcinoma cells at high IC_50_ concentration of 68 μM [6]. Unfortunately, these orthosteric Ras-GEF interaction inhibitors failed to discriminate between mutant and wild-type Ras [7]. Researchers have recently developed another series of covalent inhibitors. They irreversibly target mutant KRas^G12C^ by forming a covalent attachment to the mutant cysteine. Small molecule SM-10-70-1 is a typical example which shows antiproliferative activity with an EC_50_ of 27~47 μM [8]. The AA12 compound reduces the cell viability with an EC_50_ ≥ 0.32 μM and induces apoptosis in lung cancer cells transformed with mutant *KRas*^G12C^ [9]. However, developing effective drugs in clinical routine for *KRas*-mutated pancreatic cancer patients remains to be challenging. One of the current major challenges in oncology is to develop new dedicated strategic therapies against the traditionally “undruggable” target [7, 10, 11]. Fortunately, the increased knowledge of the complexity of the Ras signaling provides the opportunity to design better therapeutic strategy. Recently, we used 2D virtual drug screening strategy for drug-selection against the intermediate conformation of the activated KRas [12]. We searched for small molecular inhibitors capable of binding to the KRas target via *in silico* computation and selected a promising compound Spiclomazine (Supplementary Figure 1). According to the biological tests, Spiclomazine displayed potent cytotoxic activity, triggered cell apoptosis, and suppressed metastasis in pancreatic cancer cells [13]. However, its in-depth mechanism remains to be elucidated in more detail.

Here we would like to find out if the biological activity of Spiclomazine is related to the target engagement within cells. We experimentally identify Spiclomazine for the effect of the KRas-GTP-mediated target engagement on growth inhibition of pancreatic cancer cells *in vitro* and *in vivo*. In parallel, Spiclomazine is also examined in cellular thermal shift and RNA interference assays to verify our hypothesis that it directly engages KRas to a certain extent within cells. Cellular studies show the effectively reduced accumulation of activated KRas-GTP level in mutant *KRas*-driven pancreatic cancer cells, with concomitant attenuation of MEK and ERK activation. Subsequently, Spiclomazine displays survival inhibition in cancer cells by arresting cell-cycle at G2 phase without significant inhibition of survival in human normal cells, such as HEK-293 and HL-7702. Unexpectedly, mutant *KRas*-driven cancer cells are more sensitive towards Spiclomazine than wild-type *KRas* cancer cells. This shows specific biological response to cancer cells. In the *in vivo* test, Spiclomazine almost completely inhibits tumor development on MIA PaCa-2 cells-derived xenograft model in BALB/c mice through mechanism similar to what we observed in the *in vitro* experiments. From a prospective point, Spiclomazine is a specific targeted drug candidate against mutant *KRas*-driven pancreatic cancer.

## RESULTS

### Spiclomazine attenuates KRas-GTP activity and its downstream signaling

**Figure 1:**
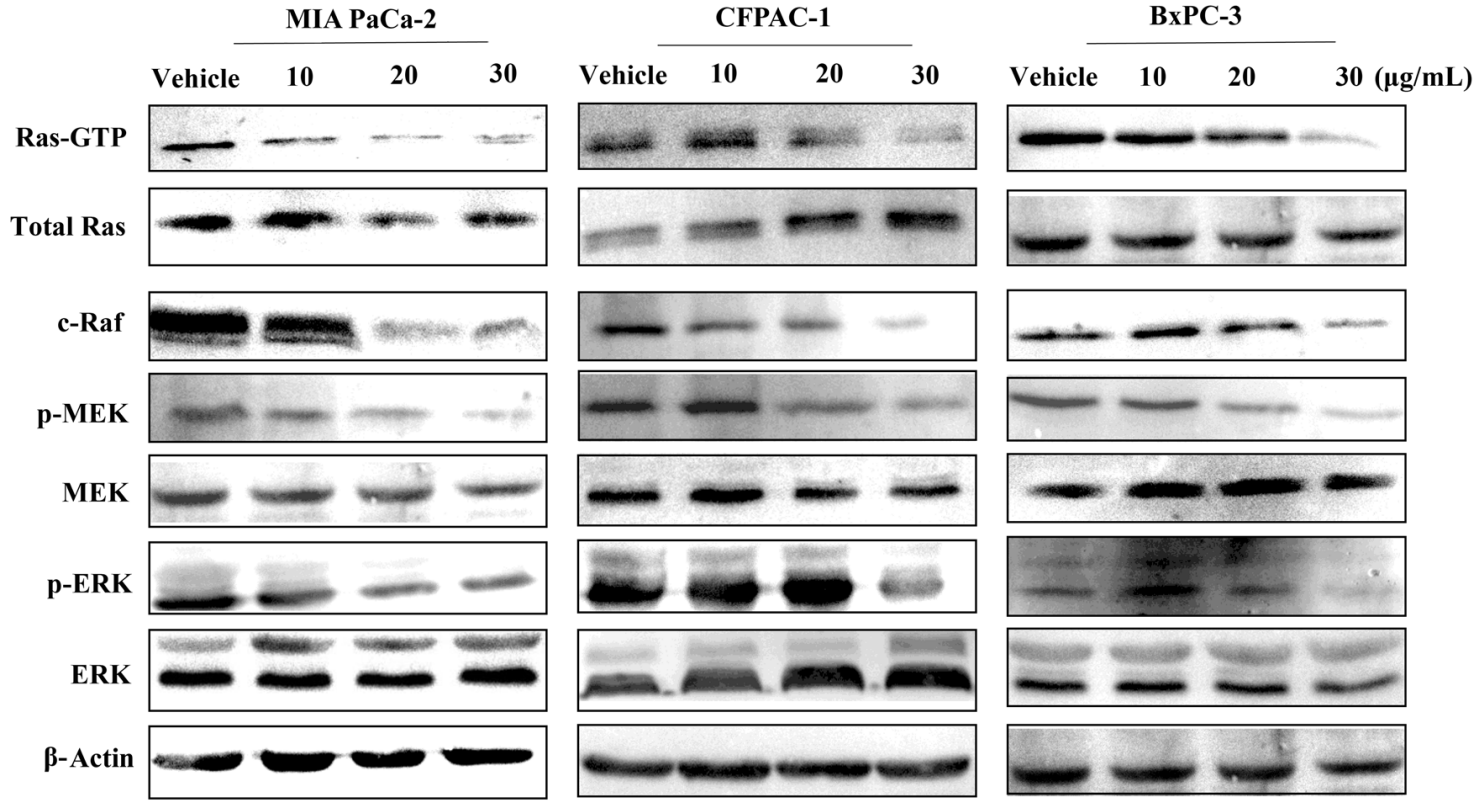
Spiclomazine attenuates Ras-GTP activity and its downstream signaling Serum-starved MIA PaCa-2, CFPAC-1 and BxPC-3 cell lines were treated with vehicle or the indicated concentrations of Spiclomazine for 24 hours, and then stimulated with EGF for 10 min before harvest. GTP-bound Ras was isolated by RBD pull-down assay and detected by Ras-GTP activation kit. Total amount of Ras was detected by anti-Ras specific antibody. Levels of c-Raf, MEK, p-MEK, ERK and p-ERK were determined by WB with specific antibody, respectively. β-Actin was used as the internal control.

In theory, Spiclomazine serves to diminish the active KRas-GTP level [12]. To account for this prediction, we first investigated the cellular effects of Spiclomazine on activated *KRas*^G12C^ (MIA PaCa-2), *KRas*^G12V^ (CFPAC-1), and wild-type *KRas* (BxPC-3) pancreatic cancer cell lines by monitoring the active KRas-GTP level. As displayed in Figure 1, Spiclomazine treatment of serum-starved MIA PaCa-2 and CFPAC-1 cell lines led to a dose-dependent and nearly complete inhibition of RAF-RBD-mediated pulldown of KRas-GTP. This observation was in agreement with our results reported in Capan-1 harboring *KRas*^G12V^ and SW1990 harboring *KRas*^G12T^ cell lines [14]. In contrast, the altered extent to KRas activation in wild-type *KRas* BxPC-3 cell line was not as strong as in mutant *KRas* cancer cell lines at the low concentrations. There were no significant changes in the expression of total Ras in MIA PaCa-2 and BxPC-3 cell lines. However, Spiclomazine treatment increased the level of total Ras in CFPAC-1 cell line when compared to vehicle-treated control (Figure 1). This can be attributed to the obvious different phenotypic characteristics. In other words, pancreatic cancer cell lines demonstrate disparate phenotypes and genotypes which are representative of pancreatic cancer sub-classes [15].

Ras activation leads to the stimulation of various signaling pathways [16]. Thus, we further detected the inhibitory effect of Spiclomazine on Ras downstream signaling. Consistent with the inhibition of active KRas activity, downstream signaling through mitogen-activated protein kinases (MAPK) pathway was inhibited by Spiclomazine in serum-starved MIA PaCa-2, CFPAC-1, and BxPC-3 cell lines (Figure 1). Notably, Spiclomazine treatment reduced the extent of c-Raf activation in MIA PaCa-2 and CFPAC-1 cell lines in a dose-dependent manner. Compared to the both mutant *KRas* cell lines, the wild-type *KRas* cell line BxPC-3 prevented the inhibition of c-Raf expression at the low drug concentrations of 10 and 20 μg/mL, but promoted the down-regulation of c-Raf at the high drug concentration of 30 μg/mL.

Quite often, the extent of activated phosphorylated extracellular signal-regulated kinase (p-ERK) was regarded as an indicator of Ras activation [17]. Based on this idea, we further detected the resulting variations of p-ERK in the cellular level. It is clear that Spiclomazine significantly reduced the extent of p-ERK in the both MIA PaCa-2 and CFPAC-1 cell lines in a concentration-dependent manner. On the contrary, BxPC-3 cell line produced more p-ERK activation at the low drug concentrations (10 and 20 μg/mL, respectively) than at vehicle control group. However, the exposure at the high drug concentration of 30 μg/mL significantly decreased the cellular p-ERK level. To quantificationally validate the observation, we simultaneously performed a flow cytometric analysis for obtaining quantificational single-cell measurements. Spiclomazine caused more inhibition of p-ERK activation at 30 μg/mL dose in mutant MIA PaCa-2 (61.57 ± 3.11%) and CFPAC-1 (57.6 ± 1.18%) cell lines than that in wild-type BxPC-3 cell line (48.17 ± 3.75%) (Supplementary Figure 2). This is in agreement with the tendency of WB analysis.

### Spiclomazine suppresses cancer cell survival

**Figure 2:**
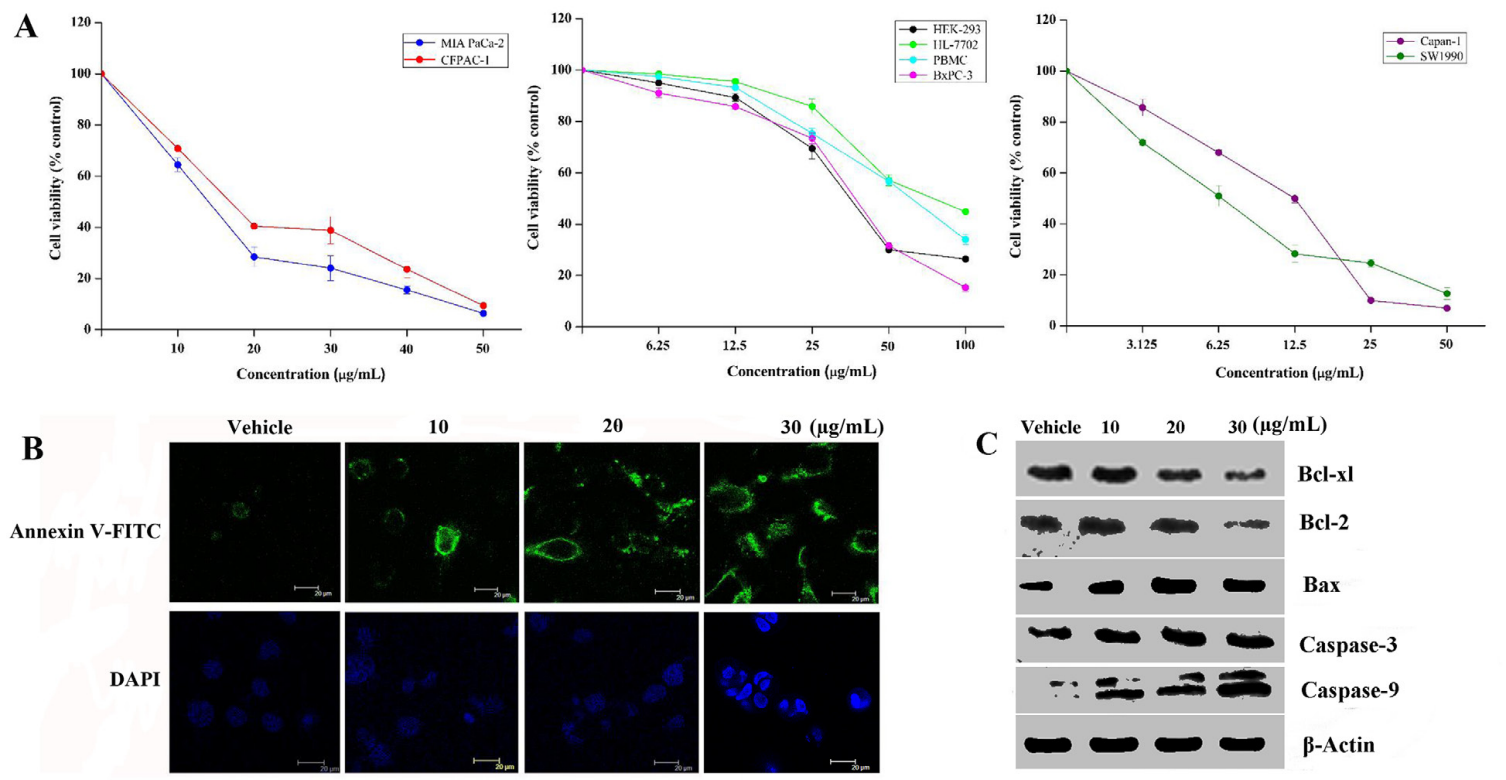
Spiclomazine suppresses cancer cell survival **(A)** Cells were treated with the indicated concentrations of Spiclomazine for 48 hours and then assessed by CCK-8 assay kit. Presented data is the mean ± SD of three independent experiments. **(B)** After 24 hours adherence, BxPC-3 cells seeded on glass-bottomed plates (100 cells/plate) were treated with the indicated concentrations of Spiclomazine for 24 hours. Then, the apoptotic Annexin V-FITC (top panel) and DAPI nuclear staining (bottom panel) images were taken by confocal-laser scanning microscope. The scale bar was 20 μm. **(C)** Effect of Spiclomazine on the expression levels of proteins related to the apoptotic pathway was examined by using WB analysis. Representative blots of respective proteins were displayed. β-Actin was used as the internal control.

Oncogenic Ras activation exploits its extensive signaling reach to affect multiple cellular processes, including cell survival and regulation of cell cycle [18]. To investigate the impact of decreasing KRas-GTP activity on cancer cell viability, we then assessed KRas specificity of viability inhibition by using a small collection of genetically annotated pancreatic cancer cell lines. The cell viability assays showed that cancer cell lines (MIA PaCa-2, CFPAC-1, Capan-1, and SW1990) harboring *KRas* mutations displayed a striking inhibition of cell survival in a dose-dependent manner (Figure 2A). The half-maximum inhibitory concentration (IC_50_) values for 48 hours are 12.9 ± 0.9 μg/mL (26.8 ± 0.9 μM) for MIA PaCa-2, 15.2 ± 2.0 μg/mL (31.5 ± 2.0 μM) for CFPAC-1, 9.5 ± 0.6 μg/mL (19.7 ± 0.6 μM) for Capan-1, and 6.8 ± 2.3 μg/mL (14.1 ± 2.3 μM) for SW1990, respectively. These IC_50_ values show a similar inhibition behavior. Comparing with the cell viability inhibition effect on cancer cell lines, Spiclomazine exhibited significantly less decrease in cell viability to non-cancerous cell lines. The IC_50_ values for 48 hours treatment are 41.9 ± 1.4 μg/mL (86.9 ± 1.4 μM) for HEK-293, 71.2 ± 3.3 μg/mL (147.7 ± 3.3 μM) for HL-7702, and 60.6 ± 2.8 μg/mL (125.6 ± 2.8 μM) for PBMC, respectively. It is worth mentioning that BxPC-3 cell line bearing wild-type *KRas* only had a modest cell viability inhibition with respect to control group. Its IC_50_ value for 48 hours (74.2 ± 0.3 μM) shows an almost 3-fold weaker cell viability inhibition than that in MIA PaCa-2 cell line harboring *KRas* mutation. Obviously, mutant *KRas*-driven cancer cell lines are markedly more sensitive toward Spiclomazine. The discriminate inhibition of the both mutant and wild-type *KRas* cancer cells is likely to avoid resulting in substantial toxicity [7]. Thus, Spiclomazine that preferentially performs its anticancer activity in activated mutant *KRas* cancer cells could circumvent this potential obstacle and are therefore particularly desirable.

The high efficacy and selectivity against cancer cells give us the motivation to investigate the underlying mechanism. As shown in Figure 2B, Spiclomazine-treated cells exhibited typically apoptotic characteristics, such as phosphatidylserine externalization and nuclear fragmentation, in BxPC-3 cells. To clarify the mechanism responsible for Spiclomazine-induced apoptosis, the expression levels of proteins in the apoptosis related pathways and the activation of Caspase-3 and Caspase-9 were experimentally examined in BxPC-3 cells (Figure 2C). Treatment of cells significantly decreased the expression levels of anti-apoptotic proteins Bcl-xl and Bcl-2, but increased the expression level of pro-apoptotic protein Bax. Simultaneously, Spiclomazine activated significantly the expression levels of Caspase-3 and Caspase-9 in a dose-dependent manner and eventually led to apoptotic death in BxPC-3 cells. The effect of Spiclomazine on apoptosis induction in BxPC-3 cells is similar to those in MIA PaCa-2 and CFPAC-1 cells [13] as well as SW1990 and Capan-1 cells [14]. This suggests a contribution of the oncogenic apoptosis induction mechanism for the cell survival inhibition.

### KRas is the main cellular target of Spiclomazine

**Figure 3:**
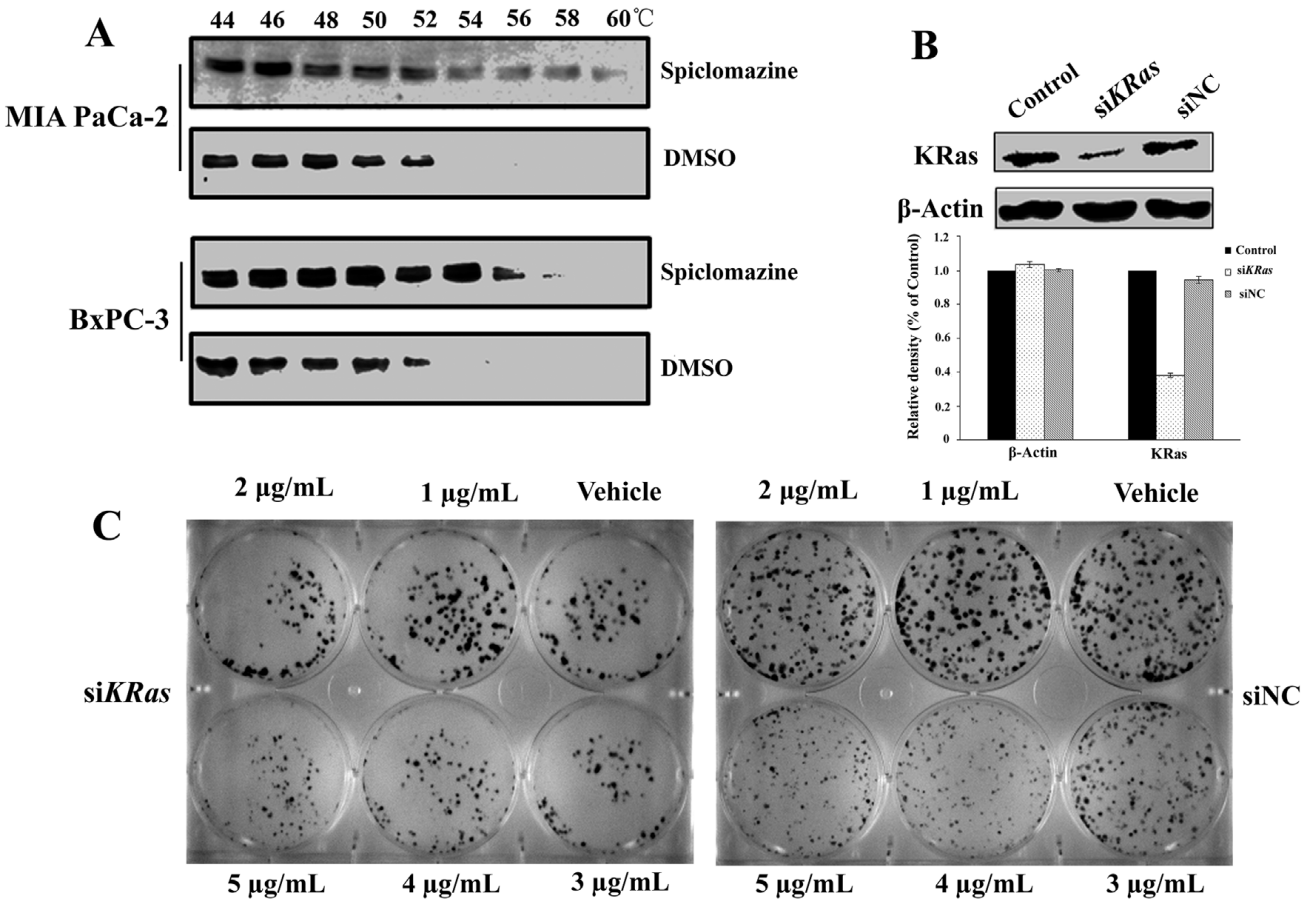
Confirmation of KRas as the main cellular target of Spiclomazine **(A)** Illustration of cellular thermal shift assay for KRas target engagement by Spiclomazine in intact MIA PaCa-2 and BxPC-3 cells. Images are representative of three independent experiments (*n* = 3) in the presence of 12.5 μg/mL Spiclomazine. **(B)** Silencing of *KRas* by siRNA. Representative western blots of three independent experiments (*n* = 3) were shown (top). Relative density of target protein was quantified and plotted (bottom). **(C)** Representative images of three independent crystal violet-stained colony formation (*n* = 3) were displayed. Silencing of *KRas* has the less extent of inhibition to the colony formation in MIA PaCa-2 cells (left). Spiclomazine significantly inhibits the colony formation of the negative controlled MIA PaCa-2 cells (right).

To rule out off-target contributed to the cell killing effect, we start monitoring whether KRas is indeed interacted with Spiclomazine in intact cells. If a cellular protein is bound by a compound, it is stabilized by the physical engagement compared to the non-engaged counterpart [19]. Based on the target engagement principle, we perform the cellular thermal shift assay (CETSA) that enables us to examine for target engagement *in vivo*. The whole-cell CETSA with Spiclomazine was analyzed for thermal stabilization of KRas in the both MIA PaCa-2 and BxPC-3 cell lines. When compared to cells treated by vehicle, Spiclomazine efficiently stabilized KRas to a certain extent with increasing the denaturation temperature (Figure 3A). Obviously, Spiclomazine more strongly interacts with and stabilizes KRas in MIA PaCa-2 (*KRas*^G12C^) cells than does in BxPC-3 (wild-type *KRas*) cells at the high denaturation temperature ranging 56~60°C. This observed difference validates the differential target engagement within cells. To further examine the specificity of Spiclomazine for KRas, *KRas* oncogene was knocked down in MIA PaCa-2 cells with specific short interfering RNA (siRNA). The number of KRas proteins was reduced in si*KRas* cells than in both control and negative control (siNC) cells (Figure 3B). In colony formation assay, Spiclomazine showed no further inhibition of or less extent of inhibition to colony formation after *KRas* knockdown when compared to siNC cells at the high concentrations of 3~5 μg/mL (Figure 3C and Supplementary Figure 4). The feeble inhibitory effect of Spiclomazine on the colony formation can be attributed to the incomplete knocked down *KRas* in si*KRas* cells. The observation strongly supports the conclusion that the cell survival inhibition caused by Spiclomazine largely depends on KRas. This further supports an on-target mechanism of action.

### Spiclomazine arrests cancer cell cycle progression

**Figure 4:**
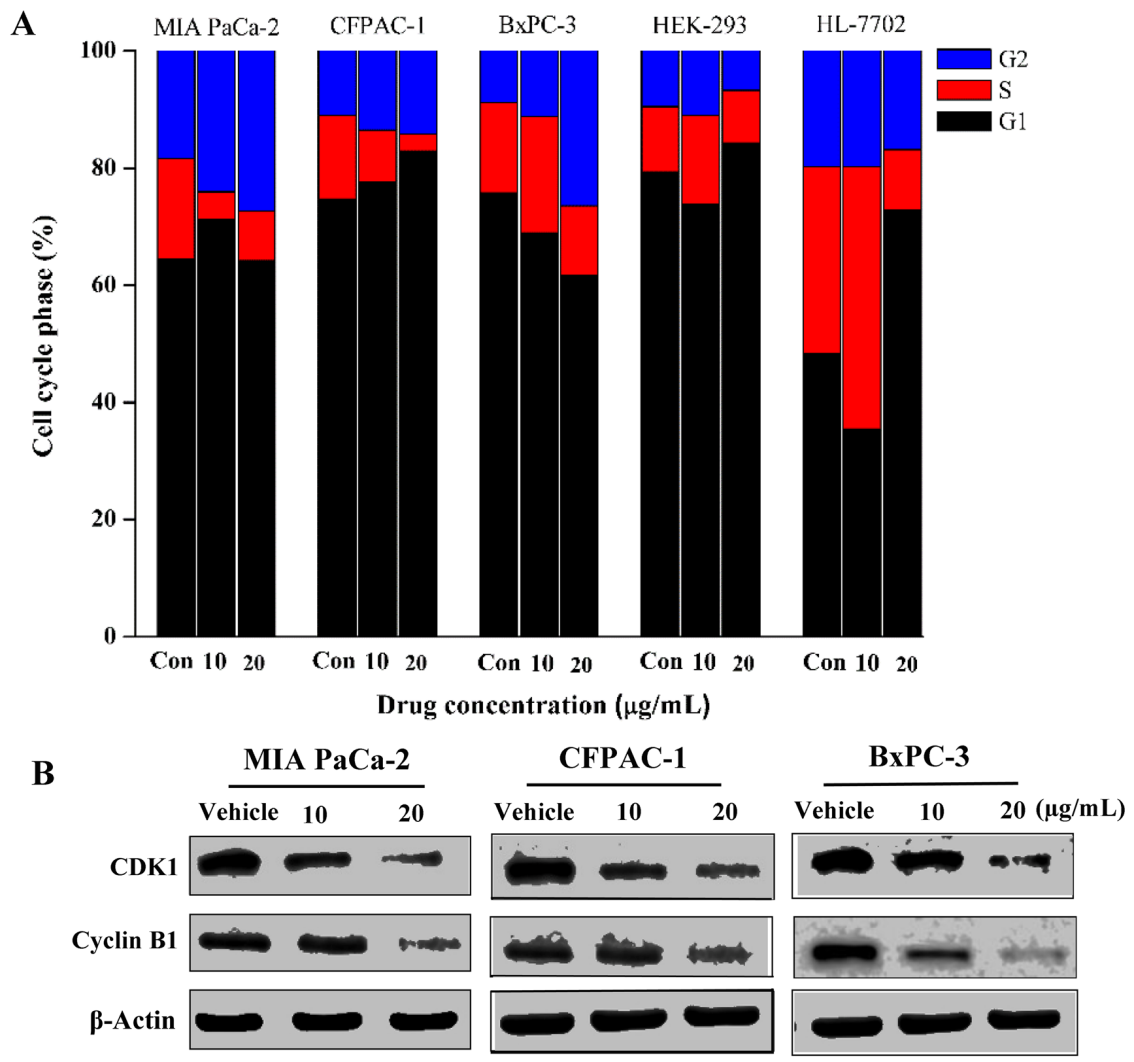
Spiclomazine arrests cancer cell cycle progression at G2 phase **(A)** The effect of Spiclomazine on cell cycle progression was analyzed in three cancerous cell lines and two human normal cell lines treated with increasing concentrations of Spiclomazine for 24 hours. **(B)** The effect of Spiclomazine on the expression levels of G2 phase-related proteins Cyclin B1 and CDK1 in pancreatic cancer cell lines were analyzed using WB assay. β-Actin was used as the internal control.

The progression of cell cycle is a complex process involving multiple gene regulations typically exhibiting G1, S and G2 phases [20]. To explore the mechanism of Spiclomazine-induced cell survival inhibition, the effect of Spiclomazine on cell cycle progression was monitored in the both human cancerous and normal cell lines treated with increasing concentrations of Spiclomazine for 24 hours (Supplemental Figure 3). All these findings implicated that Spiclomazine effectively inhibited genotype-specific pancreatic cancer cell lines at various cell cycle phases. Spiclomazine promotes cancer cell cycle arrest at either G2 phase in MIA PaCa-2, CFPAC-1, and BxPC-3 cell lines (Figure 4) or S phase in Capan-1 and SW1990 cell lines [14]. A concentration-dependent increase in the cell population at G2 phase in MIA PaCa-2 cells was observed by Spiclomazine treatment (Figure 4A). At basal level, the percentage of MIA PaCa-2 cells at G2 phase was 18.3%, while this was increased to 24.04% and 27.38% at Spiclomazine concentration of 10 and 20 μg/mL, respectively. For CFPAC-1 cells, the percentage of cells arrested at G2 phase at basal level was 10.93%. When treated by Spiclomazine with the increasing concentrations, this led to an increase to 13.56% and 14.23%, respectively. Similarly, a concentration-dependent increase in the cell population at G2 phase in BxPC-3 cells was also observed after treatment with Spiclomazine (Figure 4A). At basal level, the percentage of BxPC-3 cells at G2 phase was 8.80%, while this was increased to 11.19% and 26.43% at Spiclomazine concentration of 10 and 20 μg/mL, respectively. Unexpectedly, there is no obvious G1, S or G2 phase cell cycle arrest in human normal HEK-293 and HL-7702 cell lines under identical treatment conditions (Supplementary Figure 3 and Figure 4A). These data combined further shows selectivity and specificity in its biological response to cancerous cell lines.

To further investigate the molecular mechanism of Spiclomazine-induced cell cycle arrest, we evaluated the effect of Spiclomazine on the expression levels of Cyclin B1 and CDK1 involved in G2 phase in these pancreatic cancer cell lines for 24 hours using WB assay. As displayed in the Figure 4B, the expressions of Cyclin B1 and CDK1 displayed dramatically decreased levels in these cancer cell lines treated with Spiclomazine. Therefore, we can conclude that Spiclomazine leads to the survival inhibition that elicits a prominent, prolonged accumulation of cells at G2 phase in these pancreatic cancer cell lines.

### Spiclomazine reduces the growth of xenograft in mice

**Figure 5:**
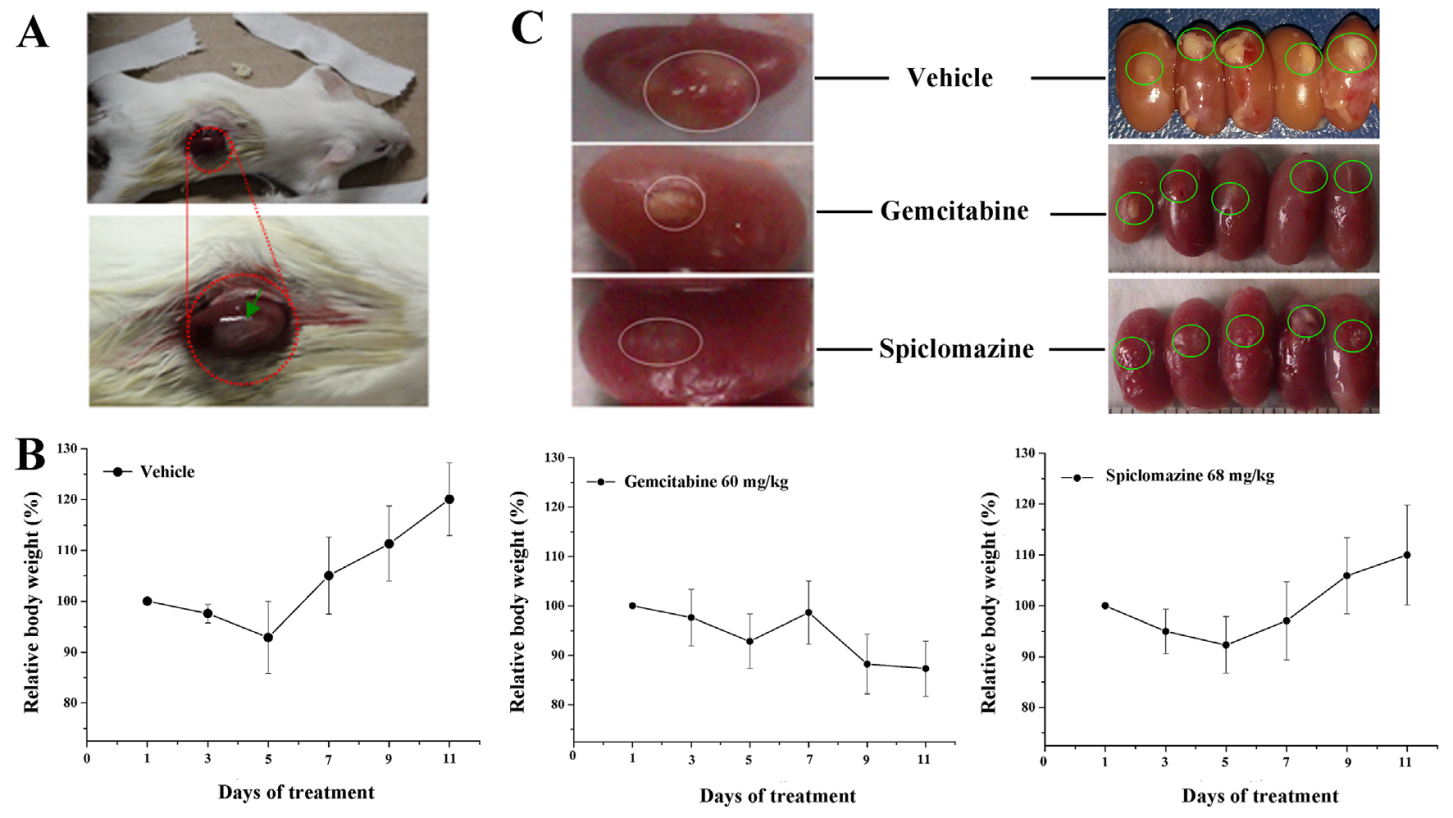
Spiclomazine exhibits *in vivo* antitumor activity in pancreatic cancer MIA PaCa-2 xenograft model **(A)** The renal capsule site (red dotted circle) was chosen for xenografting in BALB/c mice. Green arrow refers to the cells seeded into the renal capsule site. **(B)** BALB/c mice were evaluated for the potential toxicity of Spiclomazine. The experiment involved a negative control group (vehicle-treated), a positive control group (Gemcitabine-treated, 60 mg/kg), and an experimental group (Spiclomazine-treated, 68 mg/kg). Drugs were injected intraperitoneally every other day for two weeks. The body weight per mouse was recorded every other day. **(C)** Therapeutic activity of Spiclomazine in xenograft tumor BALB/c mice. Spiclomazine-based treatment significantly reduced tumor growth more effective than Gemcitabine-based treatment (indicated using white circle in left panel). In three of the five mice, the growth of tumors after Spiclomazine treatment was completely blocked (indicated using green circle in right panel).

Based on the remarkable *in vitro* potency in cellular model, we speculate that the genotype-specificity might afford a therapeutic window for the targeted inhibition of KRas in xenograft model. Aiming to determine if Spiclomazine is suitable for *in vivo* study, we first examined the induced toxic effect of the compound. For the study, we chose the renal capsule site for xenografting, as shown in Figure 5A, due to its higher graft take rate, abundant blood supply, and ability to implant a greater number of xenografts into one confined site [21]. BALB/c mice were evaluated for the potential toxicity and chemotherapeutic efficacy of Spiclomazine administered 68 mg kg^−1^ every other day for 2 weeks *via* intra-peritoneal route in the respective groups. As shown in Figure 5B, all of the Spiclomazine-treated mice did not show any body weight loss or other obvious signs of toxicity and were alive and healthy throughout this study during the treatment. By contrast, the Gemcitabine-treated mice showed a marked body weight loss and even death. Moreover, Spiclomazine-based treatment more effectively reduced tumor growth than Gemcitabine-based treatment (Figure 5C, left panel). Notably, in three of the five mice, the growth of tumors after Spiclomazine treatment was completely blocked (Figure 5C, right panel).

**Figure 6:**
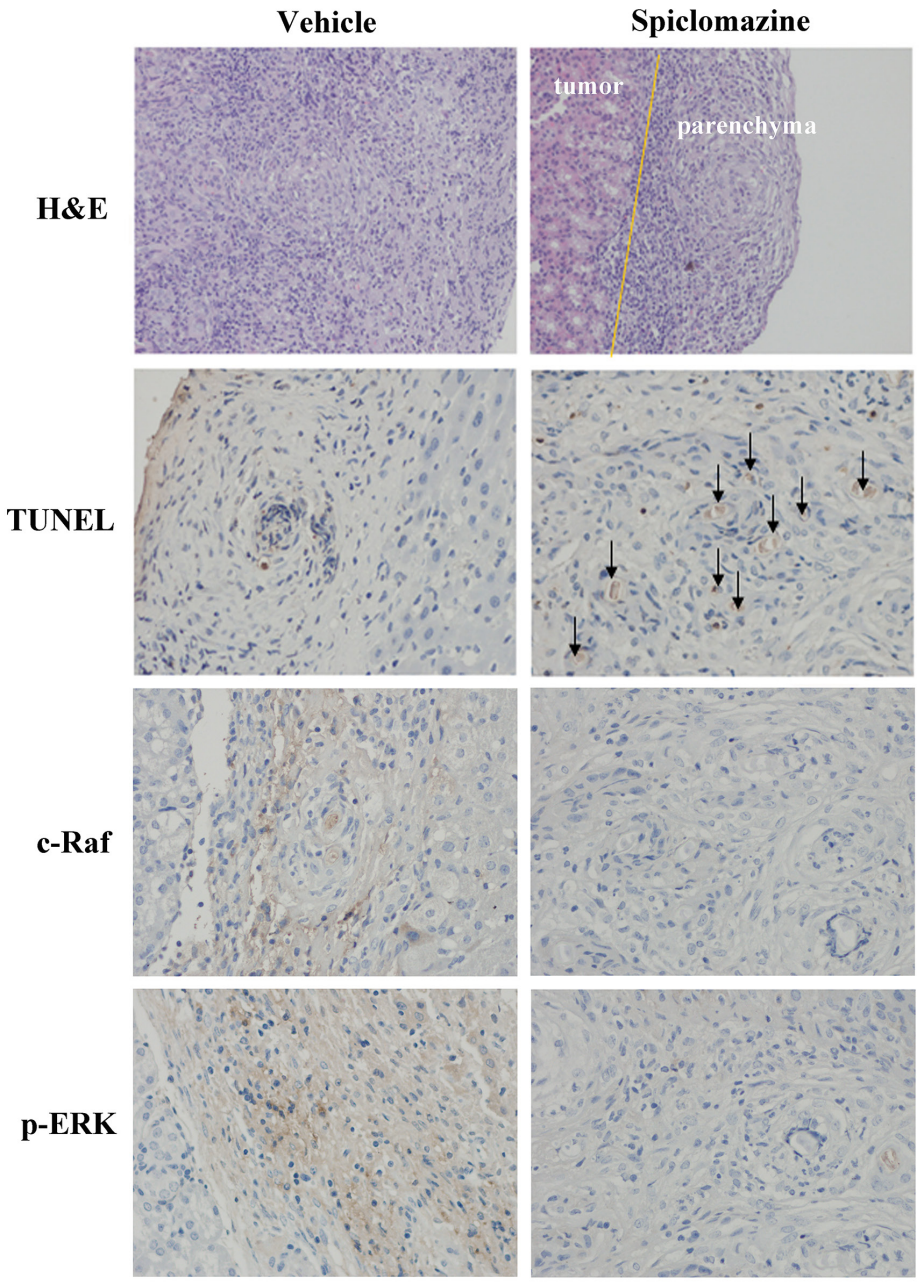
IHC analysis of tumor sections The tissue sections of the tumor-bearing kidney were evaluated by using H&E staining. A clear distinction (indicated by yellow line) between the tumor and the renal parenchyma was observed in Spiclomazine-treated groups. TUNEL assay in tumor sections showed a prominent increase of apoptotic cells (indicated using black arrow) in the Spiclomazine-treated group. Spiclomazine diminishes c-Raf protein level associated with the decreased ERK1/2 activation in xenograft tumor tissues.

Further, the tissue sections of the tumor-bearing kidneys were evaluated by using H&E staining. A clear distinction between the tumor and the renal parenchyma was observed in Spiclomazine-treated mice (Figure 6). Single cells that infiltrated the parenchyma were rarely seen and the destruction of the parenchyma was not observed. This finding supports our previous view that Spiclomazine can inhibit the migration and invasion [13]. TUNEL assay in tumor sections showed a prominent increase of apoptotic cells (Figure 6), which indicates a difference in the apoptotic rate between Spiclomazine-treated and control mice. In addition, tumor c-Raf levels from Spiclomazine-treated and control mice were compared by immunohistochemistry (IHC) analysis. Spiclomazine treatment led to a prominent reduction in tumor c-Raf level in xenograft tumor tissues (Figure 6). This is similar to the observation *in vitro* as displayed in Figure 1. Using specific p-ERK antibody, the examination of the representative tumor sections uncovered that the p-ERK level was substantially inhibited by Spiclomazine (Figure 6). These results suggest that the *in vivo* efficacy of Spiclomazine correlates with the attenuation of Ras-mediated signaling which is similar to what we observe in the *in vitro* experiments.

## DISCUSSION

Recent studies show that Ras-mediated signaling contributes to the tumorigenesis through the mutational activation of oncogenic *Ras*. Of all *Ras* mutants, mutations in *KRas* genes are major molecular abnormalities in pancreatic cancer. This is still a key issue to develop therapeutics agaist pancreatic cancer. Currently, challenges remain in the efforts to develop effectively targeted-therapic strategy for *KRas* mutants [10, 11]. The rapid advances in basic research of *KRas*-driven tumors may lead to the discovery and development of new inhibitors.

Using a structure-guided approach to target the intermediate conformation of activated Ras, we have theoretically developed Ras inhibitors *in silico* [12]. By the treatment of Spiclomazine, the Ras-GTP activity in mutant *KRas* pancreatic cancer cell lines MIA PaCa-2 (*KRas*^G12C^) and CFPAC-1 (*KRas*^G12V^) were under a significant inhibition in a dose-dependent manner (Figure 1). In contrast, the altered extent of Ras-GTP activity in wild-type *KRas* pancreatic cancer BxPC-3 cell line was not as significant as those in the both pancreatic cancer cell lines. Experimentally, the downstream effectors expression levels in mutant MIA PaCa-2 and CFPAC-1 cell lines were also under a significant inhibition in a similar manner. On the contrary, the altered extent in wild-type *KRas* pancreatic cancer BxPC-3 cell line was up-regulated at the low drug concentrations of 10 and 20 μg/mL, but down-regulated at the high drug concentration of 30 μg/mL. One seemingly plausible explanation for the observed difference is due to the generation mechanism of the intermediate conformation in different genotype-specific cell lines. The mutant KRas in cancer cells accumulates in an elevated GTP-bound proportion and thus leads to a constantly activated Ras form [22]. However, the wild-type KRas in BxPC-3 cells possesses a slow GDP/GTP exchange rate and thereby decreases the accumulation of activated Ras-GTP in a cellular context *via* a population-shift mechanism [23]. Once at the activated Ras form, the equilibrium with three different transition states is reached immediately [24]. State 1 refers to the signaling form of Ras. State 2 corresponds to the open non-signaling conformation. State 3 refers to the Ras’ conformation in complex with GEFs and effectors. This appears to be a providential opportunity for targeting the intermediate conformation and trapping it in the inactive state. If Spiclomazine is presented exactly at this time point and binds to the intermediate conformation, GTP loading will be weakened to a certain extent. The cellular level analysis suggests that Spiclomazine specifically leads to a greater reduction in the mutant *KRas* network than in the wild-type *KRas* network. These experimental results strongly support the system-level analysis [25]. In particular, it is possible that Spiclomazine at low concentration would bind to the intermediate conformation rather than other receptors due to the targeted specificity and high proportion of target receptor in mutant *KRas* cancer cells. This therefore leads to a significant attenuation of downstream signaling in mutant *KRas* pancreatic cancer MIA PaCa-2 and CFPAC-1 cells. However, there will be a low proportion of target receptor, the intermediate conformation, in wild-type *KRas* BxPC-3 cells. This is because wild-type *Ras* is predominantly GDP bound in the basal state [26]. When Spiclomazine was presented in BxPC-3 cells at low concentrations, the proportion of Spiclomazine binding intermediate conformation will be lower than those in MIA PaCa-2 and CFPAC-1 cells. This will lead to a non-specific binding of the remaining Spiclomazine to epidermal growth factor receptor (EGFR). This in turn enhances this signaling activity for the instance of up-regulation expression of c-Raf by the paradoxical activation of Ras, owing to the acute activation of EGFR [26]. This indicates that wild-type KRas can prevent the inhibition of the MAPK pathway at the low drug concentrations upon EGFR stimulation. On the contrary, oncogenic mutant KRas desensitizes cells to EGFR activation by altering EGFR trafficking, turnover, and localization [26]. With increasing the concentration of ligand Spiclomazine, the binding tendency will gradually shift to the specific binding of Spiclomazine to intermediate conformation. This therefore leads to the attenuation of c-Raf and p-ERK activation because of the positive domination of Ras signaling. We conclude that the different binding interactions and associated dynamics between Spiclomazine and the intermediate conformation are responsible for the differential effects of mutant and wild-type KRas in the MAPK pathway at the low drug concentrations. This conclusion reinforces the notion that a drug sequestering active Ras state may lead to stronger inhibition on the mutant network than on the wild-type network [25]. More importantly, CETSA and RNA interference assays provide definitive evidence that KRas is a critical molecular target of Spiclomazine (Figure 3), accounting for the underlying molecular mechanism.

The ability of Spiclomazine to decrease the amount of active Ras and to abrogate its downstream signaling in various genotype-specific pancreatic cancer cell lines suggests that this strategy is quite promising for suppressing cancer cell viability. Thus, it is important to critically assess the effect of Spiclomazine on the viability inhibition in pancreatic cancer cells. We observed the selective inhibition of pancreatic cancer cell viability with minimal effect on human normal cells at the doses we applied. By using cell-survival assays with MIA PaCa-2 (*KRas*^G12C^), CFPAC-1 (*KRas*^G12V^), BxPC-3 (wild-type *KRas*), Capan-1 (*KRas*^G12V^), and SW1990 (*KRas*^G12T^), we show that Spiclomazine treatment markedly reduces cell survival in these mutant cancer cell lines. Two plausible reasons could contribute to the observed sensibility difference. On the one hand, mutant *Ras* cancer cells are more dependent on signaling to effectors for their survival than wild-type *Ras* cancer cells [27]. This differential dependence may provide an adequate answer for the different extent to viability inhibited by Spiclomazine processing cancer cells. On the other hand, the high sensitive *KRas*-mutated cancer cells show lower Ras-GTP level and signal output after Spiclomazine treatments (Figure 1). This means that Spiclomazine displays a preferential survival inhibition effect to activated Ras cancer cells by attenuating survival signaling derived from the Ras-mediated signaling though halting activated Ras at the intermediate conformation. It is worth mentioning that the similar inhibition manner could be observed from other pancreatic cancer cell lines bearing *KRas* mutations [28], in support of the preferential survival inhibition effect on mutant cancer cell lines. In this respect, Spiclomazine locking the intermediate conformation represents a mechanism to suppress cell viability.

Moreover, Spiclomazine-treated pancreatic cancer cells exhibited cell cycle arrest (Figure 4), suggesting a contribution of inactivating *KRas* oncogene activation mechanism to the cell cycle distribution. In this study, Spiclomazine arrests the cell cycle by inhibiting the expression levels of Cyclin B1 and CDK1 involved in G2 phase in these pancreatic cancer cell lines. In Figure 4A, the G2 phase arrest in BxPC-3 cells is slightly more than those in CFPAC-1 and MIA PaCa-2 cells at the high dosage 30 μg/mL. Accordingly, the expression levels of Cyclin B1 and CDK1 are also more significantly inhibited in BxPC-3 cells at this dosage (Figure 4B). However, apoptosis is not restricted to a particular part of the cell cycle [29]. The accumulated evidences suggest that manipulations of the cell cycle may either prevent or induce an apoptotic response depending upon the cellular context [30]. Some studies reported a pro-apoptotic activity for CDK. However, the inhibition of CDK has also been shown to protect from apoptosis [31]. CDK involvement in apoptosis is cell-type-specific. However, it also depends on environmental conditions and differentiation states [32]. Based on the cell-survival inhibition results, the obvious G2 phase arrest in BxPC-3 cells may prevent an apoptotic response to Spiclomazine treatment in the cellular context of wild-type *KRas*. However, the G2 phase arrest in CFPAC-1 and MIA PaCa-2 cells may induce an apoptotic response to Spiclomazine treatment in the cellular context of mutant *KRas*. Although the cell cycle arrest was slightly more in BxPC-3 cells than those in CFPAC-1 and MIA PaCa-2 cells, the mutant *KRas*-driven cancer cells are more sensitive towards the growth inhibition by Spiclomazine treatment than the wild-type *KRas* cancer cells. The different manipulations of cell cycle to apoptosis may be attributed to the negative feedback pathways existed in the cancer cells driven by mutant *KRas* rather than by wild-type *KRas* oncogenes [26]. Although the cell-type-specific ability of cell cycle to prevent or induce apoptosis is not exactly clear in wild-type and mutant *KRas* cells, this observation has opened up a new window in the search for new therapeutic strategies to combat these diseases caused by mutant *KRas* oncogenes. These effects combined can give rise to a specific inhibition of cancer cell viability on inhibiting Ras activation. What is more important is that this preferable anti-tumor activity may be turned into specifically individualized targeted therapy for pancreatic cancer due to the up to 90% *KRas* mutations.

To ultimately evaluate the anti-tumor activity of Spiclomazine, we further extended our *in vitro* study to *in vivo* study using MIA PaCa-2 tumor xenograft as living tumor model system. BALB/c mice are immunocompetent and useful for studies on cancer. The renal capsule tumor xenograft models developed in BALB/c mice effectively circumvents the immunocompetency of the athymic nude mouse host and successfully predicts the chemotherapy responsiveness of xenografts tested in the long-term, established tumor assays [33]. The renal capsule tumor xenograft assay had been used extensively in all kinds of cancers, such as prostate cancer, ovarian cancer, melanoma, colon cancer, sarcoma, lung cancer and renal cell cancer, for the investigation of tumor response to chemotherapeutic agents [34–36]. BALB/c mice model experiments further confirmed the effective inhibition of tumor growth with minimum side effects observed. The initial toxicity study *in vivo* by the chronic injection of Spiclomazine into BALB/c mice reveals that Spiclomazine does not lead to significant alterations in body weight loss (Figure 5B). To correlate antitumor activity with the inhibition of Ras-mediated signaling by Spiclomazine observed *in vitro*, additional tumor sections were examined by section staining and IHC analysis (Figure 6). Growing under *in vivo* conditions provides sufficient evidence to support the view that Spiclomazine suppresses the tumor growth through mechanism similar to what we observed in the *in vitro* experiments. Herein, the renal capsule tumor xenograft provides a simple and reproducible method for studying carcinogenesis, motility and invasion. Thus, the BALB/c mice as the renal capsule tumor xenograft models underpin the current study. The study suggests Spiclomazine as an attractive pharmacological agent and thereby offers a unique opportunity against mutant *KRas*-driven pancreatic cancer.

## MATERIALS AND METHODS

### Ethics statement

Investigation was conducted in accordance with the ethical standards and the Declaration of Helsinki. According to national and international guidelines, the animal experiments have been approved by the Chinese Academy of Sciences Ethics Committee.

### Chemicals and reagents

Media (DMEM and IMDM) and fetal bovine serum (FBS) were purchased from Gibco (Grand island, NY). Unless otherwise notified, all chemicals were obtained from Sigma-Aldrich. Spiclomazine, 1-Thia-4,8-diazaspiro[4.5]decan-3-one,8-[3-(2- chloro-10H-phenothiazin-10-yl)propyl]-, hydrochloride (1:1) (Supplementary Figure 1), was kindly supplied from NCI/DTP Open Chemical Repository (http://dtp.cancer.gov) and further confirmed. Spiclomazine was dissolved in DMSO to make stock solution (10 mg/mL) and further diluted to appropriate concentrations with double distilled water containing 10% DMSO immediately before use. The c-Raf, MEK1/2, p-MEK1/2, ERK1/2, p-ERK1/2, Caspase-3, Caspase-9, Bcl-xl, Bcl-2, Bax, β-Actin, CDK1 and Cyclin B1 primary antibodies were purchased from Abcam (Cambridge, MA). Horseradish peroxidase-conjugated anti-rabbit and anti-mouse secondary antibodies were obtained from BD Bioscience (San Jose, CA).

### Cell culture

Human pancreatic cancer cell lines CFPAC-1, MIA PaCa-2, BxPC-3, SW1990 and Capan-1 and human peripheral blood mononuclear cell (PBMC) were obtained from American Type Culture Collection (ATCC, Rockville, MD) and cultured in DMEM or IMDM media supplemented with 10% FBS and 100 units/mL penicillin, 50 μg/mL streptomycin and 100 μg/mL amphotericin (Invitrogen, Carlsbad, CA) according to the supplier's manual instruction, respectively. PBMC was cultured according to the conditions as described in the previous publication [37]. Human normal embryonic kidney (HEK-293) and liver (HL-7702) cells were purchased from Chinese Academy of Science Type Culture Collection (Shanghai, China) and incubated in DMEM media containing 10% FBS. We started working with these cell lines 2 years ago when there was no question regarding its origin. All the cells were maintained in 25 mL flasks and kept in an atmosphere of 5% CO_2_ and 95% air under humidified conditions at 37°C. The adherent cells other than PBMC were detached from the monolayer using 0.25% trypsin and 0.53 mM EDTA for 5 min at 37°C when cells were grown to near confluence. All these cell lines were tested for mycoplasma contamination by using mycoplasma stain assay kit (Beyotime Institute of Biotechnology, China).

### Western blotting (WB) analysis

Pancreatic cancer cell lines CFPAC-1, MIA PaCa-2 and BxPC-3 were seeded onto 10 cm plates (10^6^ cells/plate). After being treated by Spiclomazine as indicated concentrations, cells harvested were washed with PBS, and then whole cell lysates were prepared by using protein lysis kit (Thermo Fisher Scientific). Equal amount of whole cellular protein (35 μg) was subjected to SDS-PAGE and the resolved proteins were transferred to polyvinylidene difluoride membrane (Upstate, Millipore). The membranes were blocked in 5% nonfat dry milk in TBST and probed for primary antibodies against the relevant proteins (1:1000 dilution). Visualization was accomplished using the appropriate horseradish peroxidase-conjugated secondary antibody (1:1000 dilution). The specific proteins were scanned by using Chemiluminescence detector (DNR, Kiryat Anavim, Israel). β-Actin (1:1000 dilution) was used for the control loading.

### Ras-GTP pull-down assay

CFPAC-1, MIA PaCa-2 and BxPC-3 cell lines were cultured in complete medium for overnight, starved for 8 hours in media containing 1% FBS and then treated for 24 hours with various concentrations of Spiclomazine. At the end of the treatment, cells were stimulated with EGF (10 ng) for 20 min. Thereafter, the cells were lysed and cell lysate was processed by Ras activation assay kit (Upstate, Millipore). The total Ras and the active Ras-GTP were detected using WB analysis as described above.

### Cell viability assay

The cell viability of eight cell lines mentioned above after being treated by double distilled water containing 10% DMSO as vehicle control groups or various concentrations of Spiclomazine as experimental groups was assessed by Cell Counting Kit-8 (CCK-8) assay (Dojindo, Japan). Each concentration of a group of drugs was set five alternative holes. The cells were treated for 48 hours and then the optical density was read with a M200 PRO NanoQuant autoreader (TECAN, Switzerland). The final concentration of DMSO in the culture media is 0.1%, which does not significantly affect the cells.

### Annexin V-FITC apoptosis assay

Based on the growth cycle of cancer cells, the cells were treated by Spiclomazine for 24 hours in order to discriminate the cell death as caused by either apoptosis induction or necrosis. Annexin V-FITC apoptosis assay was performed using a commercial detection kit (BD Biosciences, San Jose, CA). Briefly, BxPC-3 cells were seeded onto glass-bottomed plates (100 cells/plate) and cultured overnight in the incubator. Spiclomazine was added at the indicated concentrations as experimental groups, and vehicle was added as control groups. After 24 hours of treatment, cells were measured using confocal-laser scanning microscope (CLSM, TCS SP2, Heidelberg, Germany) at 488 nm to assess the FITC signals. Cells staining positive for Annexin V-FITC are undergoing apoptosis. However, cells staining negative for Annexin V-FITC are normal.

### Apoptotic nuclear staining

Spiclomazine-induced apoptotic nuclear condensation and morphological change were detected using DAPI staining kit (Beyotime Institute of Biotechnology, China). BxPC-3 cells were grown on glass-bottom plates (100 cells/plate) to 50% confluence and then cultured in the medium in the presence of various concentrations of Spiclomazine for 24 hours. Thereafter, the cells were fixed with 3.5% paraformaldehyde and then incubated in a fluid containing 2 mg/mL DAPI for 20 min. The nuclear morphology of cells was observed by CLSM.

### Cellular thermal shift assay

The ability of Spiclomazine to interact with and stabilize KRas proteins in intact cells was assessed essentially through using cellular thermal shift assay (CETSA) [19]. In short, MIA PaCa-2 and BxPC-3 cell lines were cultured in 100 mm plates (10^6^ cells/plate) to 70~80% confluency and then treated with cell media containing DMSO (final concentration 0.1%) and 12.5 μg/mL Spiclomazine for 8 hours, respectively. After treatment, cells harvested were collected by centrifugation and subsequently suspended in TBS. Thereafter, these cells were lysed on ice for 20 min and then centrifuged at 14,000 rpm for 15 min at 4°C. The cell suspension was placed into PCR tubes and heated for 3 min to 44, 46, 48, 50, 52, 54, 56, 58, and 60°C followed by cooling for 3 min at room temperature. Subsequently, precipitates were separated from the heated lysates by centrifugation at 14,000 rpm for 20 min at 4°C. The soluble protein supernatants were transferred to new microtubes and seperated by SDS-PAGE followed by WB analysis using KRas antibody (C-terminal, 1:500 dilution).

### RNA interference of *KRas*

MIA PaCa-2 cells were plated in 6-well plates for 24 hours before transfection with short interfering RNA (siRNA). A negative control siRNA (siNC) or *KRas*-targeted siRNA (si*KRas*) (Sangon Biotech, Shanghai, China) was diluted to 20 nM in RNAiMax-Lipofectamine (Invitrogen, Carlsbad, CA) containing OPTI-MEM media, and after 20 min of incubation the mixture was added drop-wise to the cells. After 72 hours incubation, cells were either lysed for immunoblotting with KRas antibody (1:500 dilution) or subjected to colony formation assay for proliferation analysis. The siNC sequence is 5’-UUCUCCGAACGUGUCACGUTT-3’ (sense) and 5’-ACGUGACACGUUCGGGAATT-3’ (antisense). The si*KRas* sequence is 5’-ACUG UACUCCUCUUGACCUGCU-3’ (sense) and 5’-CAGG UCAAGAGGAGUACAGUUA-3’ (antisense).

### Cell cycle analysis

Based on the duration of the cell cycle, it is feasible and reasonable to detect the cell cycle progression at the cell cycle checkpoint when cells are treated for 24 hours. The effect of Spiclomazine on cell cycle distribution was explored by flow cytometry. Briefly, CFPAC-1, MIA PaCa-2, BxPC-3, HL-7702, and HEK-293 cell lines were seeded in 6-well plates (10^6^ cells/well) and treated with Spiclomazine at concentration of 10 and 20 μg/mL for 24 hours, respectively. Cells were fixed in 70% (v/v) ethanol at 4°C overnight. Thereafter, cells were washed and resuspended in 1 mL PBS containing 50 μg/mL PI and 1 mg/mL RNaseA at room temperature in dark for 30 min. In total, 10,000 events were analyzed immediately in each sample by flow cytometer (Accuri C6, Ann Arbor, MI).

### Tumor growth on xenograft models

Xenografting is a powerful technique for *in vivo* evaluation of the potential of experimental cells to develop an assay with novel treatment strategies. In order to assess the toxicity and the *in vivo* efficacy of Spiclomazine, BALB/c mice as renal capsule tumor xenograft models were carried out in this study [21, 33]. In short, 10^7^ exponentially growing MIA PaCa-2 cells suspended in the fresh medium containing 10% Matrigel (BD Bioscience, Bedford, MA) were injected into the right renal capsule of each female BALB/c mouse (6~8 week old; Jilin University) according to the previous protocol [21]. Once tumor volumes reached around 50 mm^3^ on average, mice were randomly divided into three groups with five mice in each group. Group I served as negative control and received the vehicle only. Group II served as positive control and received 60 mg/kg Gemcitabine [38]. Group III received Spiclomazine intraperitoneally injected (i.p) every other day for two weeks. Spiclomazine had an antipsychotic effect and its LD_50_ (lethal dose, 50%) 3400 mg/kg was tested in mice i.p [39]. Based on the relation between the LD_50_ and the experimental dosage of pharmacodynamics, the LD_50_ value can be converted into the experimental dosage of pharmacodynamics. Quite often, the high dosage of pharmacodynamics refers to 1/5~1/10 of LD_50_, while the low dosage of pharmacodynamics refers to 1/30~1/50 of LD_50_. In this case, the low dosage of pharmacodynamics (1/50 of LD_50_), namely 68 mg/kg, was used as the experimental dosage of pharmacodynamics, due to the limitation on the amount of Spiclomazine. The body weight per mouse was recorded every other day. The mice were humanely sacrificed at the end of the second week and the kindey from each mouse was excised. A portion of the tumors was fixed in 4% (wt/vol) paraformaldehyde and embedded in paraffin for immunohistochemistry.

### Immunostaining of tissue sections

The IHC analysis was performed as previously described [40]. Fixed tumor tissues were sectioned into 4 μM thick sections using microtome (Leica Microsystems Inc., Buffalo Grove, IL). Thereafter, these sections were deparaffinized and rehydrated by washing with xylene for three times (10 min per time), 100% ethanol for two times (5 min per time), 95% ethanol for two times (6 min per time), 70% ethanol for 3 min, and 50% ethanol for 3 min. Finally, the sections were washed two times (5 min per time) in double distilled water. The sections were blocked with 6% goat serum in 1% BSA for 30 min and incubated with primary antibodies for c-Raf (1:500 dilution) and p-ERK1/2 (1:500 dilution) overnight at 4°C. After washings in PBS, the slides were stained using DAB chromogen kit (Thermofisher scientific, Fremont, CA). H&E staining was performed by using H&E staining kit (Roche Molecular Systems, Inc.). TUNEL assay was carried out by using TUNEL staining kit according to manufacturer's protocol (Roche Molecular Systems, Inc.) Images were captured with Olympus X71 inverted phase microscope (Dr. Schumann Optik OHG, Hessen, Germany) at 20× magnification.

### Statistics analysis

Data are presented as mean ± SD of triplicate experiments. Statistical analysis was conducted using SPSS 11.5 statistical software.

## SUPPLEMENTARY MATERIALS FIGURES



## Abbreviations

CDK1: Cyclin-dependent kinase 1
CETSA: Cellular thermal shift assay
ERK: Extracellular signal-regulated kinase
GTP: Guanosine-5’-triphosphate
H&E: Hematoxylin and eosin
IC_50_: Half-maximum inhibitory concentration
IHC: Immunohistochemistry
MAPK: Mitogen-activated protein kinase
siRNA: Short interfering RNA
TUNEL: Terminal deoxynucleotidyl transferase dUTP nick end labeling
WB: Western blotting.
