# Supplementary material for: Spiclomazine displays a preferential anti-tumor activity in mutant KRas-driven pancreatic cancer
Source: Oncotarget. 2018 Jan 8;9(6):6938–51. doi: 10.18632/oncotarget.24025 (PMC5805527; doi:10.18632/oncotarget.24025)
Supplement: Supplementary file 1 [file oncotarget-09-6938-s001.pdf]

Spiclomazine displays a preferential anti-tumor activity in mutant *KRas*-driven pancreatic cancer

SUPPLEMENTARY MATERIALS

Flow cytometric detection of phospho-ERK1/2

Cells after treatment as indicated in the MATERIALS AND METHODS section were fixed and permeabilized using Cytofix/Cytoperm kit (Becton Dickinson, Mountain View). After centrifugation, 0.05 µg of antibody per well in 100 µL of antibody mixture for an Alexa Fluor 488 conjugated ERK1/2 antibody (anti-phospho-p44/42 MAP Kinase, Thr202/Tyr204, BD Bioscience) was added and incubated for 1 h on ice. After washing, the samples were analyzed using fluorescence activated cell sorter FACSAria (BD Bioscience, San Jose, CA) and percentage of stained cells in each quadrant was quantified using Diva 6.0 software. In total, 10,000 events were analyzed in each sample.

Colony formation assay

The effect of Spiclomazine on the ability of MIA PaCa-2 cells treated by si*KRas* or siNC to form colonies was determined using the colony formation assay. Cells were seeded at the density of 0.5 × 10^3 cells per well in 6-well plate. Then, 24 hours later the media was replaced with fresh media containing DMSO (0.1% final concentration) or compound in triplicate. The cells were incubated for 8 days. After incubation, media was removed and cells were washed twice with PBS. Cells were fixed in 2 mL ice-cold methanol, stained with crystal violet solution (0.5% in 25% methanol) for 30 min, rinsed with tap water, and left to dry overnight at room temperature. To quantify the results, 595 nm wavelength was used to determine the ultraviolet absorbance of crystal violet following solubilisation by 70% ethanol.

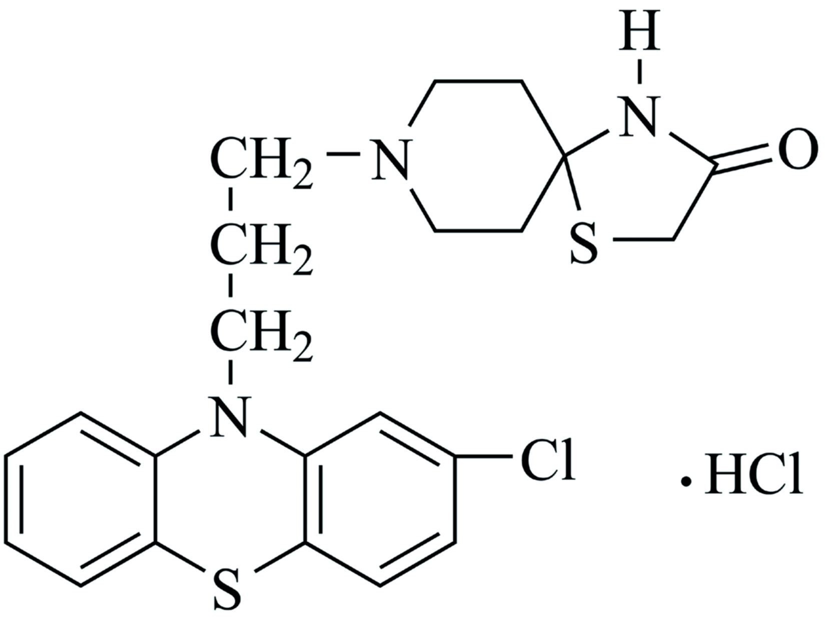

Supplementary Figure 1: Chemical structure of Spiclomazine.

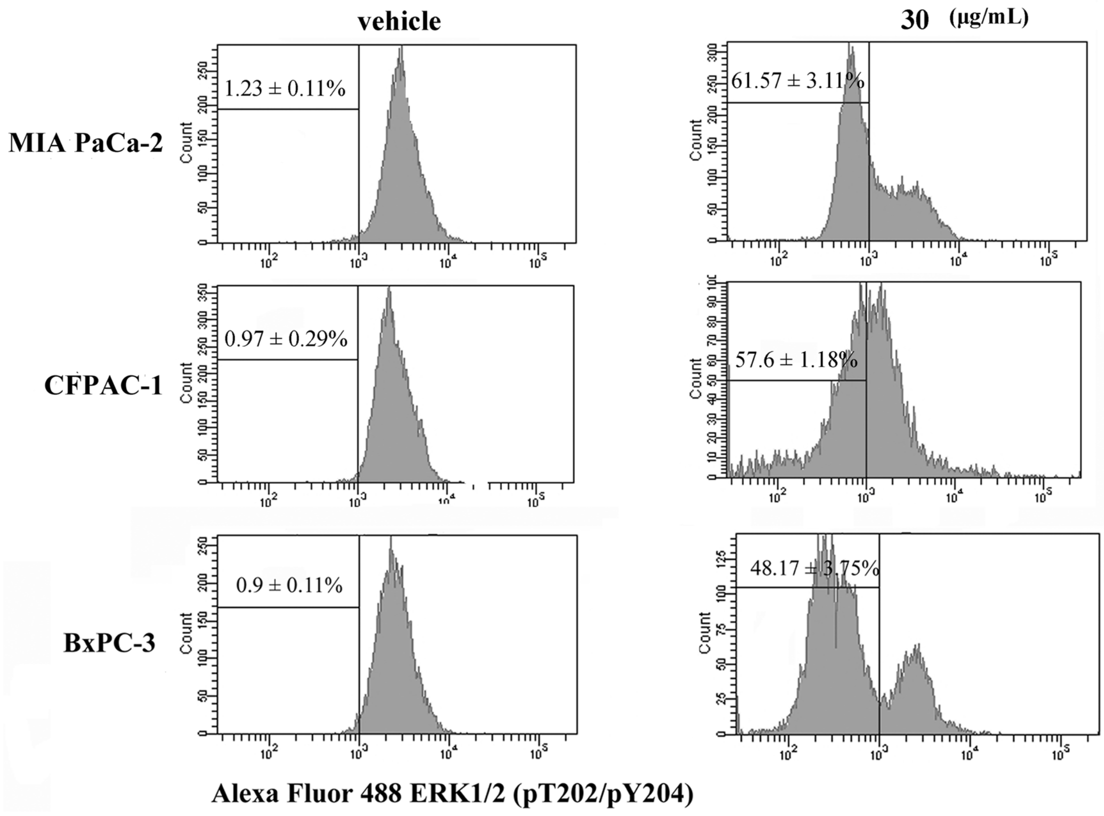

Supplementary Figure 2: Flow cytometric detection of p-ERK was performed in MIA PaCa-2, CFPAC-1 and BxPC-3 cell lines. Cells after treatments were fixed and permeabilized using Cytofix/Cytoperm kit. After washing, the samples were analyzed using flow cytometry. In total, 10,000 events were analyzed in each sample. Presented data is the mean ± SD of three independent.

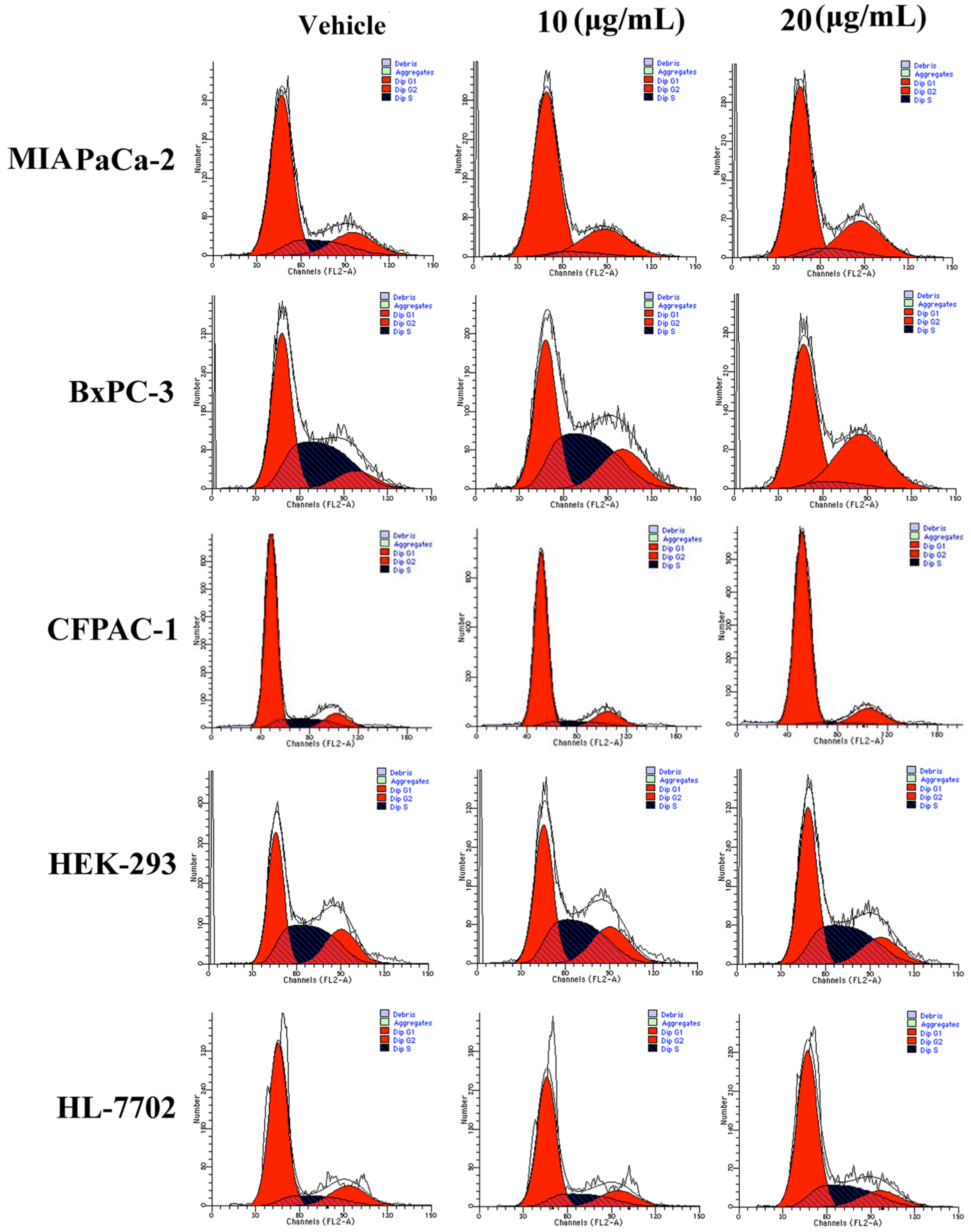

Supplementary Figure 3: Flow cytometric detection of cell cycle. CFPAC-1, MIA PaCa-2, BxPC-3, HL-7702, and HEK-293 cell lines were seeded in 6-well plates (10^6 cells per well) overnight and treated with Spiclomazine at concentration of 10 μg/mL and 20 μg/mL for 24 hours, respectively. Representative cell populations at G1, S, and G2 phases of the cell cycle were analyzed by flow cytometry. In total, 10,000 events were analyzed immediately in each sample by flow cytometer.

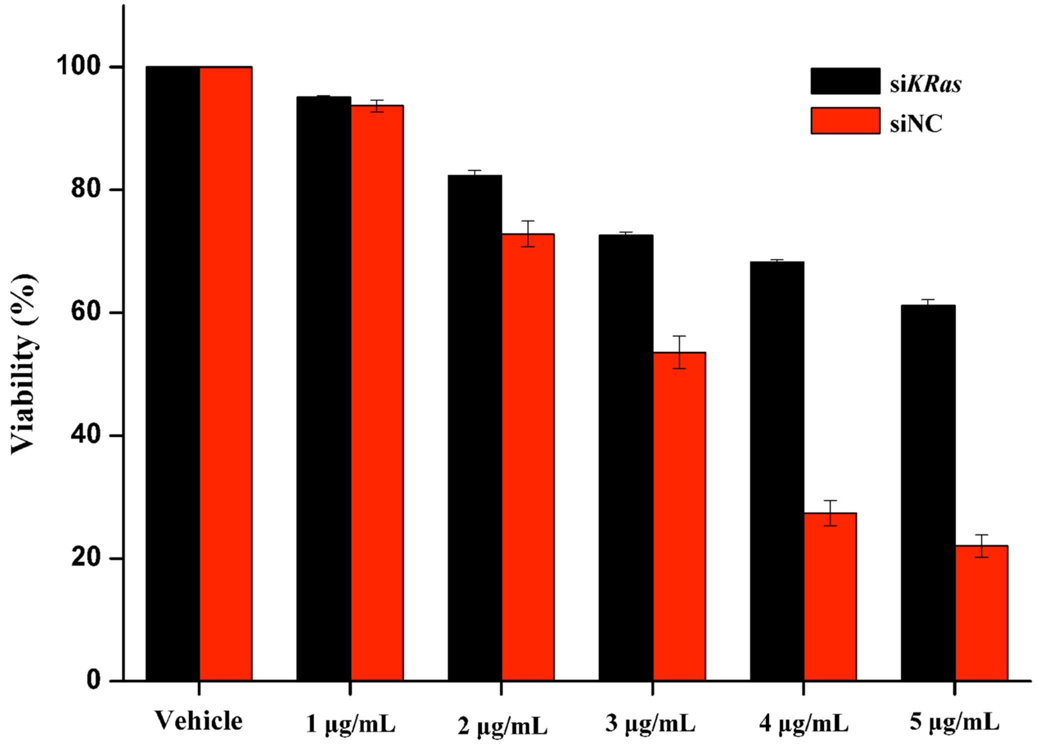

Supplementary Figure 4: KRas as the main cellular target. Stable knockdown of *KRas* by siRNA reduces the less MIA PaCa-2 cell viability in a colony formation experiment (*n* = 3).
